# Trends in the prevalence and management of pre-stroke atrial fibrillation, the South London Stroke Register, 1995-2014

**DOI:** 10.1371/journal.pone.0175980

**Published:** 2017-04-14

**Authors:** Vageesh Jain, Iain J. Marshall, Siobhan L. Crichton, Christopher McKevitt, Anthony G. Rudd, Charles D. A. Wolfe

**Affiliations:** 1 King’s College London School of Medicine, London, United Kingdom; 2 Division of Health and Social Care Research, King’s College London, London, United Kingdom; 3 National Institute for Health Research Comprehensive Biomedical Research Centre, Guy’s & St. Thomas’ NHS Foundation Trust and King’s College London, London, United Kingdom; Cardiff University, UNITED KINGDOM

## Abstract

**Background:**

Previous studies have found low use of anticoagulation prior to stroke, in people with atrial fibrillation (AF). This study examined data on patients with AF-related stroke from a population-based stroke register, and sought to examine changes in management of AF prior to stroke, and reasons for suboptimal treatment, in those who were known to be at a high risk of stroke.

**Methods:**

The South London Stroke Register (SLSR) is an ongoing population-based register recording first-in-a-lifetime stroke. Trends in the prevalence of AF, and antithrombotic medication prescribed before the stroke, were investigated from 1995 to 2014. Multivariable logistic regression analyses were conducted to assess the factors associated with appropriate management.

**Results:**

Of the 5041 patients on the register, 816 (16.2%) were diagnosed with AF before their stroke. AF related stroke increased substantially among Black Carribean and Black African patients, comprising 5% of the overall cohort in 1995–1998, increasing to 25% by 2011–2014 (p<0.001). Anticoagulant prescription in AF patients at high-risk of stroke (CHADS2 score [> = 2]) increased from 9% (1995–1998) to 30% (2011–2014) (p<0.001). Antiplatelet prescription was more commonly prescribed throughout all time periods (43% to 64% of high-risk patients.) Elderly patients (>65) were significantly less likely to be prescribed an anticoagulant, with ethnicity, gender and deprivation showing no association with anticoagulation.

**Conclusions:**

Most AF-related strokes occurred in people who could have been predicted to be at high risk before their stroke, yet were not prescribed optimal preventative treatment. The elderly,despite being at highest stroke risk, were rarely prescribed anticoagulants.

## Background and purpose

Atrial Fibrillation (AF) is a major risk factor for stroke, with a population attributable risk of 6.7% [1] and increasing the risk of stroke by up to five times in older people. [2] AF is associated with increased stroke severity, higher mortality, and increased recurrence, compared with stroke unrelated to AF. [3]

From the early 1990’s, guidelines have advocated the use of anticoagulants in patients who are at a high risk of stroke [4]; more recently, guidelines [5] have advised that antiplatelets are no longer used for stroke prevention. Nonetheless, evidence suggests that AF patients are still poorly managed. A 2010 systematic review of 54 observational studies investigating antithrombotic prescription in AF patients, [6] found anticoagulants were prescribed in only 50% of high-risk AF patients. In all but two included studies, anticoagulant prescription levels were sub-optimal (<70%) in high-risk patients, but the reasons for this remain unclear. An existing longitudinal UK study investigating the pre-stroke management of AF found that from 1994 to 2003 the proportion of patients with AF taking anticoagulants rose from 23% to 47%. The likelihood of receiving anticoagulants was greater for men, those with a higher stroke risk, and decreased sharply with age after 75 years. [7] This study adds to these findings by presenting trends over time in a multi-ethnic cohort, examining whether treatments have changed with recent updates to guidelines, and factors associated with anticoagulant use, in AF patients known to be at a high risk of stroke.

The primary aim of this study was to analyse trends in the management of AF, from 1995 to 2014, and examine factors associated with anticoagulant use, after separating AF patients into subgroups based on pre-stroke risk. Secondary aims included measuring changes in the prevalence of AF in the stroke population, and trends in the sociodemographic characteristics of those with AF-related stroke.

## Methods

The methods of the South London Stroke Register (SLSR) have been described previously. [8] The SLSR is an on-going prospective population-based register, which started in 1995, recording first in a lifetime stroke. Participants come from a defined region of Lambeth and Southwark, with a population of 591,369 according to the 2011 UK Census, with 56% white, 26% Black African/Black Carribean, and 18% other ethnic groups. [9]

Standardized criteria and multiple overlapping sources of information were used to maximize completeness of case ascertainment. Hospital surveillance of admissions for stroke included two teaching hospitals within and three outside the study area. Patients were added to the register through daily assessment of acute wards, weekly reviews of brain imaging referrals, and monthly checks of bereavement officers and bed manager records. Community surveillance of stroke included patients under the care of all general practitioners (GPs) within and on the borders of the study area (n = 147). GP’s were contacted regularly and asked to notify all new stroke patients. Additional notification sources of stroke cases included community therapists, electronic patient records, death certificates or coroner’s records as well as notification by relatives of patients.

Sociodemographic information collected including age, ethnicity, gender, socioeconomic status, deprivation and employment level. Information on family history, alcohol consumption and smoking status was also collected. Data were collected from hospital records and by contacting the patient’s usual general practitioner on risk factors which had been diagnosed prior-to-stroke, and any regular prescribed medication.

Data comprised binary (yes/no) information on a range of conditions including AF, cardiac failure, hypertension, diabetes, TIA and peripheral vascular disease. These data were collected through General Practice and hospital records. Similarly, past medication history was recorded to determine whether patients were prescribed antiplatelets, anticoagulants, or any other medication.

### Statistical analysis

STATA 13 was used for all analyses. The significance of trends over time (from 1995 to 2014) in socio-demographics, AF prevalence and prescription rates were analysed using the χ^2^ test, and Kruskal-Wallis test for comparisons between more than two groups. Risk of stroke was estimated using the CHADS_2_ score, a clinical tool used to estimate future risk of stroke in AF patients.

Logistic regression models investigating the odds of anticoagulant prescription were conducted in the overall AF cohort, as well as within high-risk (CHADS_2_ score ≥2) patients. The following variables were included in the multivariable analyses: age, ethnicity, gender, year group of stroke, alcohol consumption, deprivation, past TIA, uncontrolled hypertension and hemorrhagic stroke. Ages were grouped as: under 65, 65–74, 75–84 and over 85 years old. Ethnic groups included in the regression models were white and non-white, to ensure each group had sufficient numbers of patients for analysis.

When investigating factors associated with the pre-stroke management of AF, hemorrhagic stroke is a potential source of confounding, which was adjusted for. It is likely that if the patient had a hemorrhagic stroke, they may have had bleeding risk factors prior to stroke, unrecorded by the SLSR, but known to the physician, which may have affected the patient’s management. For this reason, a sensitivity analysis was conducted in which the multivariable logistic regression model was run only on ischemic stroke cases, after excluding hemorrhagic strokes. Calculated odds ratios for this model (data not shown) was very similar to those from the model presented in this paper which included all types of stroke, but adjusted for stroke type.

Additional risk factors for bleeding which may alter the management of AF are given by the HAS-BLED score. [10] Although the dataset did not include all the variables needed to allow calculation of full HAS-BLED scores for each patient, the prevalence of four HAS-BLED variables: alcohol (≥8 units), uncontrolled hypertension, age (>65), and medication predisposing to bleeding (NSAIDs) was recorded. It was found that very few patients were prescribed an NSAID. For this reason, medication predisposing to bleeding was been excluded from the multiple logistic regression, while the remaining three variables were adjusted for.

## Results

A total of 5041 stroke patients were registered from 1995–2014, with 816 (16%) of these diagnosed with AF prior to stroke. 478 (59%) were female. Median age was 80 years, with an interquartile range of 72 to 86. The majority of AF patients were white (685, 84%). In total 72 strokes in AF patients were hemorrhagic (9%), 629 (77%) were ischemic and 115 (14%) were unclassified. Trends in the sociodemographic details of the study population and prevalence of AF are presented in Table 1. There was a significant change in the ethnic profile of AF patients with just 5% black in 1995–1998, increasing to over 25% in the period 2011–2014 (p<0.001). Age and gender profiles stayed relatively similar over time, with the majority of AF patients being female, and the median age of AF patients at the time of their stroke being 80 years.

**Table 1:** AF patients in the SLSR—Sociodemographic and Lifestyle Characteristics from 1995–2014, and changes over time.

		1995–1998 (%)	1999–2002 (%)	2003–2006 (%)	2007–2010 (%)	2011–2014 (%)	Total (%)	P (Trend)
**SLSR**	Patients with AF (%)	252 (20.7)	138 (13.9)	148 (15.4)	133 (15.0)	145 (19.9)	816 (16.2)	0.342
**Gender**	Male	103 (40.9)	52 (37.7)	63 (42.6)	53 (39.9)	67 (46.2)	338 (41.4)	0.346
	Female	149 (59.1)	86 (62.3)	85 (57.4)	80 (60.2)	78 (53.8)	478 (58.6)	
**Age**	<65	25 (9.9)	18 (13.0)	21 (14.2)	18 (13.5)	29 (20.0)	111 (13.6)	0.144
	65–74	54 (21.4)	23 (16.7)	29 (19.6)	25 (18.8)	22 (15.2)	153 (18.8)	
	75–84	91 (36.1)	57 (41.3)	61 (41.2)	51 (38.4)	51 (35.2)	311 (38.1)	
	85+	82 (32.5)	40 (29.0)	37 (25.0)	39 (29.3)	43 (29.7)	241 (29.5)	
**Ethnicity**	White	238 (94.5)	117 (84.8)	127 (85.8)	109 (82.0)	94 (64.8)	685 (84.0)	<0.001
	Black Caribbean	8 (3.2)	8 (5.8)	11 (7.4)	9 (6.8)	22 (15.2)	58 (7.11)	
	Black African	4 (1.6)	2 (1.5)	4 (2.7)	5 (3.8)	15 (10.3)	30 (3.7)	
	Other	2 (0.8)	11 (8.0)	6 (4.1)	10 (7.5)	14 (9.7)	43 (5.3)	
**Smoking**	Yes	98 (38.9)	36 (26.1)	61 (41.2)	42 (31.6)	41 (28.3)	278 (34.2)	0.401
	No	100 (39.7)	73 (52.9)	46 (31.1)	53 (39.9)	62 (42.8)	334 (41.0)	
**Alcohol**	Yes	163 (64.7)	57 (41.3)	73 (49.3)	52 (39.1)	43 (29.7)	388 (47.8)	<0.001
	No	89 (35.3)	69 (50.0)	58 (39.2)	61 (45.9)	77 (53.1)	354 (43.6)	
**Stroke subtype**	Ischemic	192 (76.2)	121 (87.7)	128 (86.5)	109 (82.0)	79 (54.5)	629 (77.1)	0.796
	Hemorrhagic	23 (9.1)	11 (8.0)	14 (9.5)	15 (11.3)	9 (6.2)	72 (8.8)	

The prevalence of AF decreased (from 21%) in the 1995–1998 period, to approximately 14% in the following years (1999–2002). From 1999–2002 to 2011–2014 there was a statistically significant increase in AF prevalence from 14% to 20% (p = 0.003).

### Prescribing of antiplatelets and anticoagulants

Overall, 147 (19%) of all patients with AF were prescribed an oral anticoagulant. The anticoagulants prescribed were warfarin (135 patients [92%]) and dabigatran (1 patient), with 11 patients (7%) on an unspecified anticoagulant. 346 patients with AF (44%) were prescribed an antiplatelet; these comprised monotherapy with aspirin (296 patients [86%]), clopidogrel (19 patients [6%]), dipyridamole (10 patients [3%]); or combination therapy (16 patients [5%] clopidogrel + aspirin, 1 patient clopidogrel + dipyridamole). The antiplatelet class was unknown for four patients. Eighteen patients (2%) were prescribed both an antiplatelet and an anticoagulant.Table 2 reports the demographics of AF patients and their prescription, by their estimated risk of stroke.

**Table 2:** Difference in demographics and prescribed antithrombotic treatment by pre-stroke CHADS2 score.

		CHADS2<2	CHADS2≥2	P -value
**Gender**	Male	145 (52.5%)	193 (35.7%)	0.005
	Female	131 (47.5%)	347 (64.3%)	
**Age**	<65	71 (25.7%)	40 (7.41%)	<0.001
	65–74	98 (35.5%)	55 (10.2%)	
	75–84	61 (22.1%)	250 (46.3%)	
	85+	46 (16.7%)	195 (36.1%)	
**Ethnicity**	White	230 (83.3%)	455 (84.3%)	0.264
	Black Caribbean	18 (6.5%)	40 (7.4%)	
	Black African	15 (5.4%)	15 (2.8%)	
	Other	13 (4.7%)	30 (5.6%)	
**Prescription**	Anticoagulant	46 (17.3%)	83 (15.7%)	<0.001
	Antiplatelet	83 (31.2%)	245 (46.5%)	
	Both	2 (0.8%)	16 (3.0%)	
	None	135 (50.8%)	183 (34.7%)	

#### Management of high-risk AF patients

**Fig 1:**
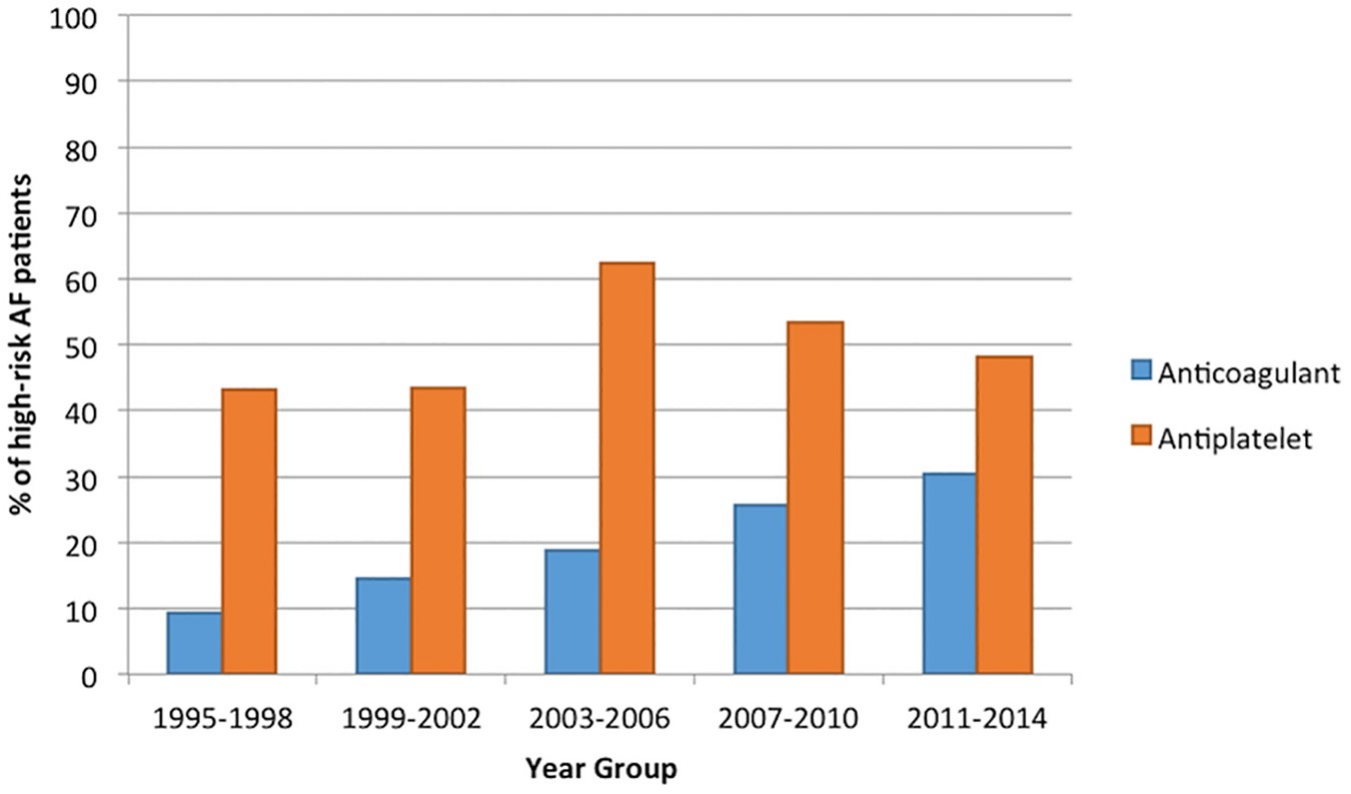
Trends in the management of high-risk AF patients (CHADS_2_≥2).

Fig 1 demonstrates trends in the management of AF patients at high-risk of stroke, according to the CHADS_2_ score. Anticoagulation rates in the high-risk group increased with time (p<0.001). 9.3% were prescribed an anticoagulant in 1995–1998, increasing to 14.5% in 1999–2002 and steadily further until 30.4% in 2011–2014. From 1995 to 2014 there was no significant trend in antiplatelet prescription (p = 0.143), which ranged from 43.0% in 1995–1998 to 62.4% during the period 2003–2006. Since then, whilst antiplatelet prescription rates remained noticeably higher than anticoagulation rates, they fell to 53.3% in 2007–2010, and 48.04% in 2011–2014.

#### Management of low-moderate-risk patients

**Fig 2:**
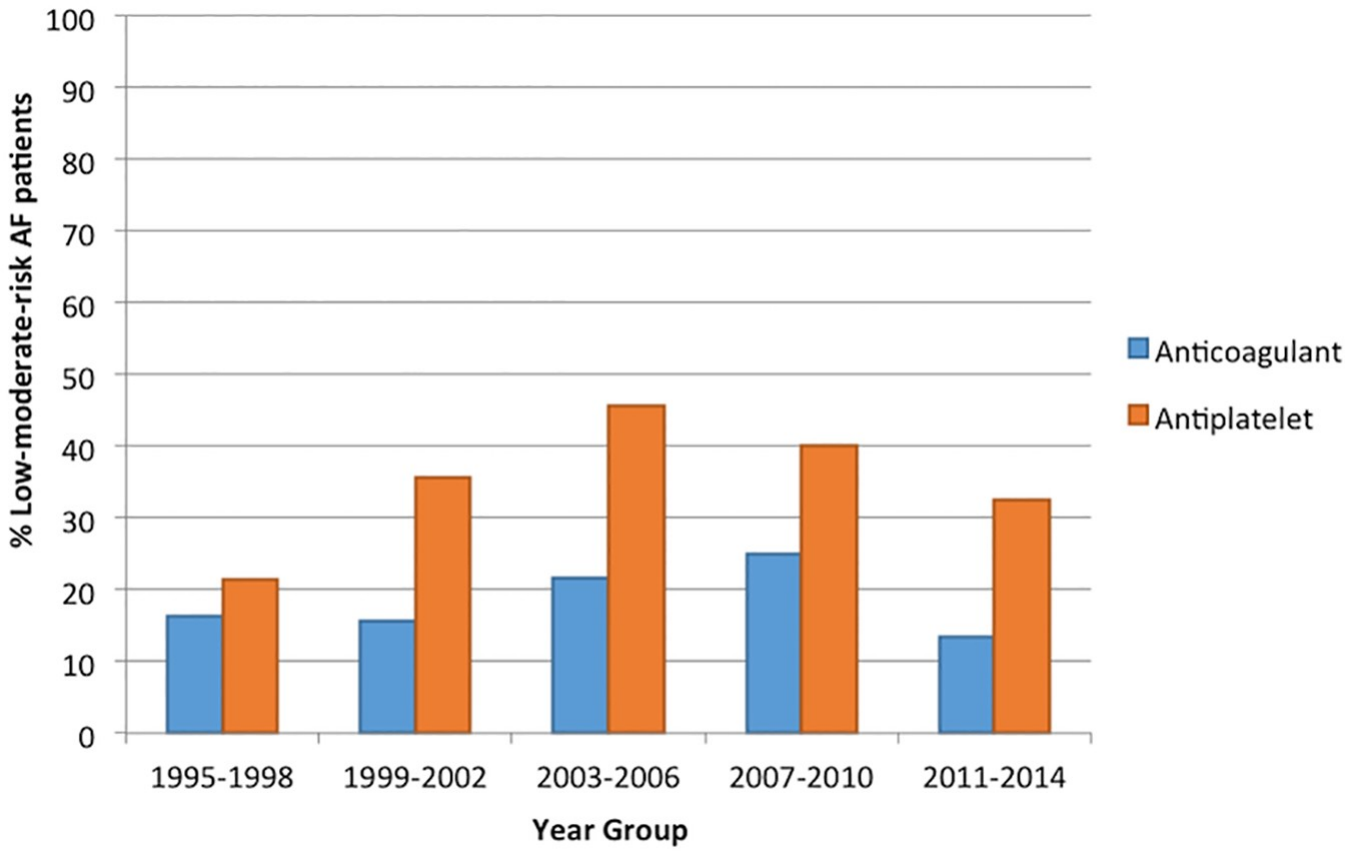
Trends in the management of low-moderate-risk AF patients (CHADS_2_<2).

276/816 patients with AF had a CHADS2 score <2. Changes in the management of AF patients considered at low-moderate-risk of stroke can be seen in Fig 2. Antiplatelets were more readily prescribed in all year groups, showing significant changes over time (p = 0.027), peaking at 45.7% in 2003–2006 and then decreasing. Although numbers were low, prescription of anticoagulants showed no clear trend over time (p = 0.669) in this group.

#### Prevalence of HAS-BLED factors

Of all AF patients, 234 (28.7%) had hypertension but were not on medication for this, and of these 198 were at a high-risk of stroke. Most of the AF cohort (705 patients) were over 65 years old. In the high-risk group, 500 patients (93% of the entire high-risk group) were over 65 and in the low-moderate risk group 205 patients were over 65 (74.3% of the low-moderate risk group). In total 178 AF patients (21.8%) consumed ≥8 units of alcohol per week. This included 102 patients at high-risk of stroke (18.9% of the high-risk group) and 76 at low-moderate risk (27.5% of this group). Only seven AF patients were prescribed an NSAID, six of whom were categorized as high-risk. NSAIDs included in the analysis were ibuprofen, diclofenac, naproxen, celecoxib, mefenamic acid, etoricoxib and indometacin.

### Predictors of AF management

#### Univariable logistic regssion–all AF patients

In the overall AF population, unstratified by pre-stroke risk, increasing age was negatively predictive of anticoagulant prescription. Those of a non-white ethnicity were approximately 57% more likely to be prescribed an anticoagulant, compared with their white counterparts (OR 1.57, 95% CI 0.99–2.47). In addition, patients who drank eight or more units per week were almost half as likely to be prescribed an anticoagulant compared to those who drank less (OR 0.52, 95% CI 0.32–0.85). Those on anti-hypertensive drugs were significantly less likely to be prescribed an anticoagulant (OR 0.69, 95% CI 0.48–0.99).

#### Multivariable logistic regression–anticoagulant prescription

In the overall AF cohort anticoagulant prescription was more than twice as likely in 2011–2014, compared with 1995–1998 (OR 2.8, 95% CI 1.15–4.14). Older patients between the ages of 65 and 74 (OR 0.33, 95% CI 0.17–0.63), as well as those over 85 years (OR 0.19, 95% 0.1–0.37) were significantly less likely to be prescribed an anticoagulant, compared with those under 65. We found a 44% decrease in likelihood of anticoagulant prescription in patients who drank eight or more units per week (OR 0.56, 95% CI 0.31–1.03); however the differene was not statistically significant.

Table 3 reports the factors associated with anticoagulation within the high-risk group of AF patients. There was a significant and linear increase in anticoagulant prescription with time, more pronounced an increase than in the overall AF cohort. The odds of a high-risk patient being treated with an anticoagulant were three times higher in 2011–2014, compared with 1995–1998 (OR 3.01, 95% CI 1.15–7.89). The analysis also revealed a non-linear relationship between age and anticoagulant prescription, with those aged 75–84 being more likely to receive an anticoagulant than patients aged 65–74 or 85 and above. Patients aged 75–84 were approximately 63% less likely (OR 0.37, 95% CI 0.16–0.88), those aged 65–74 were 84% less likely (OR 0.16, 95% CI 0.05–0.52) and those aged 85+ were 88% less likely (OR 0.12, 95% CI 0.05–0.33) to be prescribed an anticoagulant, compared with those under 65. Ethnicity, gender, deprivation, previous TIA and uncontrolled hypertension appeared to have no correlation with anticoagulant prescription in either high-risk, or overall AF groups. Hemorrhagic stroke was associated with greatly increased odds of anticoagulant prescription throughout all analyses. Sensitivity analyses based on a partial CHA_2_DS_2_-VASc score [11] (excluding data on previous thromboembolism) did not find any qualitative change from the main analysis (S1 Fig).

**Table 3:** Factors associated with anticoagulant prescription in high-risk patients (CHADS_2_≥2); results of multivariable logistic regression analysis.

Variable	Odds Ratio	95% confidence interval	P-value
Age<65	1.00	-	0.001
Age 65–74	0.16	0.05–0.52	
Age 75–84	0.37	0.16–0.88	
Age 85+	0.12	0.05–0.33	
White ethnicity	1.00	-	-
Non-white ethnicity	1.24	0.60–2.53	0.562
Female	1.00	-	-
Male	0.71	0.40–1.25	0.234
Time of stroke 1995–1998	1.00	-	0.008
Time of stroke 1999–2002	1.69	0.67–4.29	
Time of stroke 2003–2006	2.18	0.93–5.10	
Time of stroke 2007–2010	2.84	1.22–6.62	
Time of stroke 2011–2014	3.01	1.15–7.89	
Alcohol <8 units/week	1.00	-	-
Alcohol ≥8 units/week	1.04	0.51–2.11	0.921
Carstairs Score of Deprivation	0.97	0.90–1.04	0.356
No previous TIA	1.00	-	-
Previous TIA	1.46	0.78–2.71	0.236
No/controlled hypertension	1.00	-	-
Uncontrolled hypertension	1.54	0.85–2.80	0.156
Ischemic stroke	1.00	-	-
Hemorrhagic stroke	8.74	4.22–18.09	<0.001

## Discussion

### Key findings

From 1999 to 2014 the prevalence of pre-stroke AF in this cohort of stroke patients increased, as did the proportion of AF-related strokes occurring in ethnic minority patients. In high-risk patients anticoagulant prescribing significantly increased over the entire study period (1995–2014), although rates remained low at just 30% in the period 2011–2014. Antiplatelets were more frequently prescribed in all time periods. Increasing age was strongly associated with non-prescription of anticoagulants; this was found not only in the very elderly, but consistently in all age groups over the age of 65. Ethnicity, gender, and lower socio-economic status were not associated with anticoagulant prescription.

### Trends in the prevalence of AF in a stroke population

A previous SLSR analysis (until 2010) found that the prevalence of AF diagnosed before stroke reduced from 20.6% (1995–1998) to 14.9% (2007–2010). [12] However, this analysis has found that this trend did not continue; the prevalence of AF diagnosed before stroke increased from approximately 15% in 2007–2010 to just fewer than 20% in 2011–2014. Possible explanations for this increase include better detection of AF, and an aging population in the study area. [13] Black African and Black Caribbean ethnicities now make up a larger proportion of AF-related strokes, increasing from approximately 4% and 7% (2007–2010) to 10% and 15% respectively (2011–2014). The prevalence of AF in black stroke populations has not been widely reported; most of the literature on the epidemiology of AF is based on predominantly white populations in North America or Europe. [14] This recent increase in black people with AF may simply reflect increasing average age of these ethnic groups in Lambeth and Southwark. The Black Caribbean 60+ population is projected to grow by 59%, (from around 5,000 to 8,200) and the Black African 60+ population by 164% (from 2,300 to 6000), from 2011 to 2031. [13]

### Predictors of suboptimal management

High-risk AF patients aged 85 and over were 88% less likely to be prescribed anticoagulant therapy compared with those aged under 65. A 2012 meta-analysis of 28 observational studies from the US similarly found a reduced likelihood of warfarin prescription with every ten-year increase in age (OR 0.78, 95%CI 0.68–0.90). [15] One possible reason for older patients not receiving warfarin could be clinician or patient concern with an increased risk of intracranial hemorrhage, which increases most markedly at 85 years of age. [16] Evidence from the 2007 BAFTA trial [17] (n = 973) suggests such fears may be unjustified, since the yearly risk of bleeding events was similar between warfarin (1.4%) and aspirin (1.6%).

In the high-risk group 65–74 year olds were 84%, and 75–84 year olds 63%, less likely to be given an anticoagulant compared with those under 65. As patients aged, they were prescribed warfarin more, until patients reached a more advanced age (85+ years), at which point warfarin use reduced once again. It seems therefore that the relationship between age and warfarin use is complex in nature, based heavily upon balancing of stroke and bleeding risks.

### Strengths and limitations

The key strengths of this study include a long (20-year) ongoing period of data collection, a dataset with a wide range of variables allowing the calculation of pre-stroke risk scores, and unlike many existing studies [15] an ethnically diverse study population. Data were collected in a protocol driven, standardized fashion with previous analysis of the SLSR estimating completeness of case ascertainment at 88%. [18] Finally, even with a full clinical assessment, not all stroke patients would be identified has having high stroke risk in advance of their stroke (due to limitations in risk prediction tools). By using risk data collected before the time of stroke, we were able to assess the quality of prescribing on the basis of information which would reasonably be available to a clinician considering primary prevention.

One of the foremost limitations of this analysis was that we were unable to calculate a full HAS-BLED score for each patient, and therefore fully ascertain bleeding risk, (as a high risk of bleeding may have led to an anticoagulant not being prescribed). However a 2011 Danish study found that the benefits of anticoagulation were likely to outweigh the risk of harm even in patients at the highest risk of bleeding (HAS-BLED ≥ 3). [19] Paradoxically this group may stand to obtain the largest benefit, since many factors which increase bleeding risk likewise are associated with increased stroke risk (note that age, hypertension and past stroke are included in both CHADS2 and HASBLED scores). Secondly, data on adherence to medication were not collected. However, a prospective US cohort study of warfarin users found that among 145 participants the mean per cent of days of non-adherence to warfarin (for the prevention of stroke) was 21.8%. [20] A sub-optimal level of patient adherence may exaggerate the negative effect of a pre-existing low rate of warfarin prescription. Thus trends in the management of AF and factors associated with inappropriate management may hold even greater importance after adherence to medication has been accounted for. Thirdly, the results from this analysis cannot be generalized to the wider population with AF, since this study included stroke patients only. Patients on warfarin would be less likely to have a stroke, and therefore be included in this study, compared to those on antiplatelets. So, although analyzing only those who had a stroke did provide a truly high-risk sample of AF patients, the rates of anticoagulant prescription found here may be lower than in the overall AF population.

### Implications for clinical practice

Qualitative research has found that many patients have specific concerns with anticoagulant use, including widespread awareness of the historical use of warfarin as rat poison. [21] Howitt and Armstrong found that patients who did not feel they were unhealthy or at obvious risk of disease, refused to take warfarin. [22] Other factors associated with refusal of warfarin include the need for regular blood tests to monitor anticoagulation control, the time taken to do the tests, and needing to abstain from alcohol. [21] Physician barriers to prescribing warfarin include the perceived risk of bleeding, the likelihood of patient compliance, and potential drug interactions or medical contraindications. [21,23]

As there are such numerous barriers to acceptance of warfarin, sharing decision making with patients may lead to conflict with guideline recommendations. [24] Thomson et al [25] conducted an RCT comparing computerized decision aids versus a doctor-led treatment recommendation in AF patients deciding about whether to take warfarin. It found that only 25% of people decided to start warfarin in the shared-decision making group, compared to 94% in the doctor-led advice arm. Although it is possible that patients can be reassured to some extent by better information around anticoagulation; the studies described here suggests that patients have valid concerns and might be making informed decisions to avoid anticoagulation.

### Implications for future research

This study found a large increase in AF-related stroke occurring in non-white ethnic groups. The CHADS_2_ and CHA_2_DS_2_-VASc scores were developed in predominantly white populations. [26,27] Given differences in stroke pathophysiology, which have been demonstrated in different ethnic groups, [28] better evidence is required of whether standard management strategies are effective across ethnic populations.

With such low rates of anticoagulant prescription found in high-risk AF patients, it is important to consider the use of alternative forms of therapy. A 2012 meta-analysis of 12 RCTs found that NOACs (including dabigatran, rivaroxaban, apixaban and edoxaban) were associated with an overall reduced rate of stroke compared with warfarin, including a reduced risk of bleeding. [29] In the SLSR just one patient received a prescription for a NOAC (dabigatran etexilate), in 2014. This may be because similar to many areas of the UK, GPs in the South London area are not allowed to initiate NOAC treatment. [30] There are potential barriers to the prescription of NOACs. Firstly, given these are relatively new medicines, evidence is still emerging about which groups will benefit most; indeed there have been reports of thrombotic complications in certain populations. [31] Secondly, the twice-daily dosing schedules of some NOACs may be more difficult for patients to adhere to than a daily regimen. Finally, bar dabigatran, antidotes are still being developed to reverse the anticoagulant effect of NOACs, particularly limiting their use in patients with co-morbidities. [32] Despite these current limitations, in future, NOACs may replace warfarin as a first-line therapy for patients with AF. It will therefore be essential to investigate whether factors found to be predictive for suboptimal management in this study, are overcome under new treatment regimens.

## Conclusions

From 1999 to 2014, AF-related stroke increased and affected more people in ethnic minority groups than ever before. Although the rate of antithrombotic prescription has been increasing, most patients diagnosed with AF before their stroke were not prescribed any treatment. Despite longstanding evidence that anticoagulation is superior to antiplatelet treatment for stroke prevention, antiplatelets were the most frequently prescribed treatment. This pattern did not change over the study period, and was consistent even in those with multiple diagnosed vascular risk factors (who could have been identified as being at high risk before their stroke). Anticoagulant prescribing was exceptionally low in older people, the group who stand to have the largest benefit. Novel anticoagulants might prove more acceptable to patients, but use may still be restricted by prescribing policies and relatively limited clinical experience.

## Supporting information

S1 Fig Factors associated with anticoagulant prescription in high-risk patients (CHA_2_DS_2_-VASc≥2); results of multivariable logistic regression analysis. (DOCX)
