# Supplementary material for: Trends in the prevalence and management of pre-stroke atrial fibrillation, the South London Stroke Register, 1995-2014
Source: PLoS One. 2017 Apr 14;12(4):e0175980. doi: 10.1371/journal.pone.0175980 (PMC5391932; doi:10.1371/journal.pone.0175980)
Supplement: S1 Fig — (DOCX) [file pone.0175980.s001.docx]

S1 Fig - Factors associated with anticoagulant prescription in high-risk patients (CHA_2_DS_2_-VASc≥2); results of multivariable logistic regression analysis

| Variable | Odds Ratio | 95% confidence interval | P value |
| --- | --- | --- | --- |
| Age<65 | 1.00 | - | - |
| Age 65-74 | 0.230 | 0.10-0.52 | <0.001 |
| Age 75-84 | 0.478 | 0.25-0.93 | 0.029 |
| Age 85+ | 0.157 | 0.07-0.34 | <0.001 |
| White ethnicity | 1.00 | - | - |
| Non-white ethnicity | 1.165 | 0.67-2.03 | 0.590 |
| Female | 1.00 | - | - |
| Male | 0.876 | 0.56-1.37 | 0.559 |
| Time of stroke 1995-1998 | 1.00 | - | - |
| Time of stroke 1999-2002 | 0.904 | 0.42-1.96 | 0.798 |
| Time of stroke 2003-2006 | 1.400 | 0.71-2.78 | 0.336 |
| Time of stroke 2007-2010 | 2.070 | 1.06-4.05 | 0.034 |
| Time of stroke 2011-2014 | 2.609 | 1.31-5.18 | 0.006 |
| Alcohol <8 units/week | 1.00 | - | - |
| Alcohol ≥8 units/week | 0.625 | 0.32-1.21 | 0.164 |
| Carstairs Score of Deprivation | 0.963 | 0.91-1.02 | 0.173 |
| No previous TIA | 1.00 | - | - |
| Previous TIA | 1.537 | 0.90-2.61 | 0.112 |
| No/controlled hypertension | 1.00 | - | - |
| Uncontrolled hypertension | 1.088 | 0.67-1.76 | 0.731 |
| Ischemic stroke | 1.00 | - | - |
| Haemorrhagic stroke | 7.422 | 4.07-13.53 | <0.001 |
